# The Dental Hygiene Council of Massachusetts

**Published:** 1910-02-15

**Authors:** William H. Potter


### THE DENTAL HYGIENE COUNCIL OF MASSACHUSETTS.

BY WILLIAM H. POTTER, D.M.D.

This council originated from committees appointed by the following societies: Harvard Dental Alumni Association, Harvard Odontological Society, American Academy of Dental Science, Metropolitan District of Massachusetts Dental Society, and Boston and Tufts Dental Alumni Association. The committees thus appointed formed the "Dental Hygiene Council of Boston," and the first meeting was held December 23, 1907. In June, 1908, the council was enlarged by members appointed by the Massachusetts Dental Society. Its name was then changed to "The Dental Hygiene Council of Massachusetts;" and it consisted of twelve members.

The objects of the council can be stated under the following heads:

(a) Publication and distribution of literature on oral and dental hygiene.

(b) Urging the work of examination of teeth of school children.

(c) Giving advice to educators and others as to practical methods of promoting dental hygiene.

(d) Assisting in the formation of dental dispensaries, where dental services can be had at a nominal cost.

(e) Establishing lists of young practitioners who are willing to work within the means of persons of small wages.

(f) Calling attention to the way in which the condition of the teeth enters into the problem of the general health and physical development of the community.

The work actually accomplished under these headings, to date, can be briefly stated.

(a) The following publications have been originated or adopted by the council:

1. "Care and use of the Teeth." (A tract.)

2. "Dental Hygiene Council of Massachusetts: Its Purposes, Its Work, Its Need." (A tract.)

3. "The People's Disease: How to Prevent it." (Pamphlet.)

4. "Dental Caries: A Factor in Disease." (Pamphlet.)

5. "The Teeth of Public School Children: How Can They Be Improved?" (Pamphlet.)

6. "Oral and Dental Conditions in Tuberculosis." (Pamphlet.)

7. "How to Care for the Mouth and Teeth." (Pamphlet.)

8. (In preparation) Dental Hygiene Wall Chart for School Room Use.

(b) In urging the examination of the teeth of school children, it has been the endeavor of the council to create public sentiment with the expectation that public sentiment would result in a public demand for dental examinations.

(c) In giving advice as to practical methods of promoting dental hgyiene, we have made use of our published literature, and also have written many personal letters explaining details.

(d) In the matter of dispensaries the council has advocated the formation of dispensaries in all localities where they did not exist, and where the local practitioners were desirous of maintaining them. The council has helped in planning the work of several dispensaries in the State.

(e) The council has not yet established a list of young practitioners, but expects soon to do so as there is a frequent demand for such men, and it is evident that much help to any community can be provided in this way.

(f) The relation between the teeth and problem of general physical development has been effectively brought out by a Dental Hygiene Conference which was held January 18-23, in Boston.

At this conference educators, health officers, social service workers and dentists conferred together and the importance of dental hygiene was emphasized in a way that made a decided impression on the community.

(g) A Dental Hygiene Exhibit was one of the first things established by the council. It demonstrated cardinal facts about the teeth and mouth; showed the origin and progress and decay; indicated how decay could be minimized or prevented. This exhibit has been shown five times in Boston, and once in the following places: Salem, Lynn, Fall River, Quincy, Gardner, Worcester, Brockton, Providence, R. I., and Hartford, Conn. It has been engaged for Albany, N. Y.

It has traveled with the exhibit maintained by the Boston Association for the Relief and Control of Tuberculosis, and been recognized as an important part of that exhibit.

(h) Lectures on dental hygiene, especially designed for school children, their parents and teachers, have been given upon invitation by members of the council. The number of these lectures amounts to at least thirty in the year and a quarter of the council's existence. The demand for these lectures has been encouraging, and the interest in them very satisfactory.

The public awakening with regard to dental hygiene means much for the profession. Dr. Jessen, of Strasburg, has said: "It is time for the dentists to emerge from his office where he has been content to faithfully repair the effects of dental caries, and take his part in maintaining the general health of the community."

This means an increased regard for the profession in the community. The more closely we touch the problem of life and death, the more importance will be conceded to our work.

Endowment of dental schools and dental dispensaries will surely follow when the public realizes the importance of a clean and healthy mouth.

DISCUSSION.

Dr. Herbert L. Wheeler:—While the subject before the meeting is being discussed, I want to give a short history of the beginning of this work. As far back as 1902 or 1903, what was, as far as I know, the first free dental clinic in America was established in connection with St. Bartholomew's Clinic, of New York City. Dr. J. Morgan Howe was the organizer of this movement, and I think I was the first one to offer my services in the clinic. There were about ten or fifteen young men who served there regularly every afternoon and evening in the week, except Saturday. Owing to unfortunate circumstances, it came about that the change of rectors of St. Bartholomew's Church was the cause of the appointment of a man in charge of the clinic who had no standing in the profession. It became necessary for the men to quit the magnificent work that they had done at this clinic, where nearly three thousand patients were treated yearly.

The value of this work so impressed itself upon some of us that I went about New York City for a year or two trying to find some one that would support a clinic of this kind. Finally I came in contact with Dr. William H. Allen, who was at that time the agent of the Society for Improving the Condition of the Poor. Through his good offices, Mr. Charles Loring Brace, through the Children's Aid Society, furnished the necessary means to equip a dental clinic, besides giving us a room in one of the industrial schools of this society. The equipment was partly obtained by donation, Dr. Louis Nash donating a chair, and some other things being donated in this way. The first free dental clinic, which was really the forerunner of this dental hygiene movement, was this one established in New York City. This clinic is still running, the number of patients attended to being yearly between five hundred and six hundred.

This attracted so much attention that demands for dental clinics have become very general. The Children's Aid

Society is anxious and willing to equip every one of its twenty-two schools with dental clinics, furnishing the room, equipment and supplies, if the dentists can be found to do the work.

At Bellevue Hospital, one of the hospitals supported by the city, a dental department has been already organized, where we have, at the present time, about twenty men, who were appointed as visiting and attending dentists, the same as the surgeons and physicians are appointed. This clinic has been so satisfactory that the Board of Directors of Bellevue and the allied hospitals are willing and anxious to establish dental departments in some three or four other hospitals, equipping and furnishing supplies as soon as the men can be secured. Some charitable institutions have awakened so to the necessity of the care of the teeth that they are willing and ready to pay either a fee for every visit or a regular yearly salary for a dentist to attend to the children under their charge.

This, briefly, is a history of the work which called attention to the dental needs of the children of this country. The work was very much assisted by the Committee of Physical Welfare, which was established about 1905 to 1906. This committee not only looked upon the question of the teeth, but the general physical condition of the children in the public schools of New York City. They found that seventy-five per cent. of the children attending public schools in New York City had never been in a dentist's office; and if the proportion of children who need dental services were in the same ratio throughout the country, there were, at least, 12,000,000 children in the United States in need of dental services.

From the enthusiasm of the young men working at the Children's Aid Dental Clinic, which is now in charge of Dr. A. H. Merritt, has undoubtedly sprung this present wide-awake movement and a realization of the necessity of active measures for preventive dentistry.

If we are going to do anything in this line we have got to do as they have done in Boston, and it requires public-spirited men to do it. It would be something if, instead of staying in the office all day, we give one afternoon a week or monthly to this work, but the dentist has not been educated to it. I say this not as a criticism, but it is an unfortunate fact that the dentist of the United States, despite his boast of superiority, has not been educated to any extent as a professional man, and the professional men of Germany are vastly superior to us in the matter of ideas of professional obligation, despite the fact that we may be superior in the technical work.

Drs. Potter and Woodbury and various other able men of Boston are struggling against great odds to bring this matter to the attention of the proper persons and in securing the assistance they need, and this is the difficulty we find in New York, and it is the young men who are doing the work. There is always an excuse on the part of the older men—no doubt about that—but suppose the physicians did that way! You know from what I have said that I appreciate that the dentist cannot do what the physician does, nevertheless, the dentist can do something. He can spend a half a day a week or month, and he can cut out that time, on his appointment book if he wishes to. The dentist, if he is anything at all of a success, can take some time to himself. If he wishes to go to a base ball game he has little trouble in getting out for an afternoon, and you will find it just as easy to acquire the habit of going to the hospital or dental clinic as to close the office for some other reason.

By the superior advantages that have been given us in acquiring a dental education, we are under obligations to the public in this age, and it is not right or proper for us to be found in a position that we were years ago.

The public is now awakening to the necessity of the physical welfare of the community, and of the units that make up the community. Prof. Sedgwick has recently called it the "call of public health;" and it is absolutely necessary that the dentist shall fill in his small part of the circle. The physicians have done us the courtesy to leave the mouth entirely to us, and we haven't grasped the situation. The mouth is the field of the dentist, and he is expected to attend to it, and if the dental profession does not awake to its obligation it will pay the penalty, and if the colleges do not awaken to the obligations that they have to the young men they will pay the penalty. Every teacher must awaken to the fact that they are there for some purpose other than to teach you to fill a few cavities in the teeth, and if they don't awaken to this we must have teachers who will.

The Board of Health of New York City has examined one hundred and seventy-two thousand children this year, and seventy to eighty per cent. were found to be in need of dental services. Of six hundred thousand children in the public schools of New York City, seventy-five per cent, have never been to a dentist, with the exception possibly of having a tooth extracted now and then. If all the dentists of America were to emigrate to New York for the purpose of repairing these defective teeth in a given number of years, the number of dentists would have to be increased tremendously to accomplish the task.

Philadelphia has stood in the vanguard of the profession for years, and I am simply astonished at what you are not doing here. I graduated from a college in this city, and I think a great deal of Philadelphia, but you are standing still. You are just marking time. That is all you are doing over here and you ought to be ashamed of it. It is time you got busy and did something worthy of Philadelphia and of what it has done in the past. If Philadelphia does not do her duty, and Boston and New York do not do their duty, and Chicago does not do its duty, there will be nothing done, and I hope after what Dr. Potter has said here to-night that you will take hold and do something along this line, and you can do it just as well as they do in Boston or New York. It is an untried field, to be sure, and you must expect to make mistakes. We all make mistakes, and we must forget that sometimes the physicians' opinions do not suit us, and we will have to stop condemning physicians because they don't know a whole lot about the mouth, for there is a whole lot about other parts of the body that we don't know. It is not such a lot of work to give a little time each week, and I hope you gentlemen will take hold and do your part in this work.

Dr. S. H. Guilford:—Perhaps we have not done anything in a very large way, but Dr. Wheeler makes a mistake, as many other people do sometimes, in assuming things of which he has no knowledge. He said that if the colleges do not do their duty they will hear of it; in other words, he intimated that the colleges were doing nothing in this matter. I believe our colleges are doing something. I know they are. Every day, in the college with which I am connected, there is something more done than examining children's teeth. After examination the teeth are cared for and put in proper condition. Such service cannot be extended very greatly by any one institution, but our college has for some time taken entire charge of the teeth of the children of one charitable institution gratuitously. We have also signified our willingness to co-operate with the Bureau of Health in any organized movement to care for the teeth of the poor children of our city. I know that other dental colleges here have likewise tendered their services. What should be done toward meeting the demands of the large number of public school children in a systematic way, and what will be done, we cannot exactly say. It is a problem which will have to be met. At present we cannot reach all, but we do reach some of them. We shall probably have the same trouble that they have had in New York in getting enough dentists to be willing to do this work.

Dr. I. N. Broomell:—I spoke to Dr. Wheeler before he opened the discussion, and said that in Philadelphia we have no dentist who is doing the work that he and Dr. Potter appear to be doing in New York City and Boston.

Afterward I recalled the fact Dr. Guilford has referred to—that the school which I represent is at the present time giving services to thirty school children each week—ten children three days of each week—which means quite a number when you sum it up for the whole year. This work began last fall, and is being carried out the present session.

There is no doubt that there are many children who ought to have the attention of the dental profession in this generous way. I cannot, however, agree with all of the opinions of Dr. Wheeler. In the first place it seems to me that many of these children should be brought into contact with their family dentist first, and in that way be initiated into what we expect in the way of tooth preservation.

I would state that the thirty children treated each week are free of charge, both as to service and material.

DR. JOSEPH HEAD:—I think and I hope that the time is not far distant when the dentists of Philadelphia will combine and form a public clinic. I for one shall be glad to take part in such a movement. What we have done with reference to the dental colleges, I cannot now say, but we do know that in general dental colleges do give work inferior to that of regular practitioners They have the wor of students, and, of course, the work of the students is not as good as the work of dentists. In that respect we are inferior to the medical men, who give their very best work. Many of the dental colleges, if I am not mistaken, do get a certain amount of compensation for each individual filling, and I don't know just how much charitable work they do, but when I went to the dental college I know the patients paid, and while it was less than they would pay to the professional dentist, since the students were all paying for the privilege of working on the patients, in the aggregate we can hardly say it was charity work.

DR. H. E. ROBERTS:—I think I have never heard the public school problem presented as clearly as it has been to-night by both Dr. Wheeler and Dr. Potter. Philadelphia is noted for being slow, but I think it is gradually waking up to the fact that something must be done. The other morning a gentleman, who is very widely connected with charities, asked me how he could secure some one who would take care of patients in his private hospital. He wanted some one he could call on, and he said he was willing to pay some small amount, so that there would be nothing lost by it.

When the men who are the leaders in the public school, and when the city fathers, or councilmen, also become interested in the fact, or wake up to the fact, that it is advisable to have the children's teeth looked after, then I think means will be provided to accomplish that end, but I doubt very much if the dentists of their own efforts can force that. It must, I believe, come through the councilmen, through the people who are interested in children, particularly. Get them interested in it, and let them start the movement; then if the dentists are willing to come forward and do their part, I feel that that is the way by which we will arrive at something, but I doubt if we will succeed in much if the dentists try to take the lead. I think we ought to do everything we can to awaken interest in the public Mind, and everything done in that line is so much gained. I would like to thank Drs. Potter and Wheeler for their papers and discussions this evening.

Dr. Wheeler:—I want to correct my old friend and teacher, Dr. Guilford, that is, his interpretation of what I said or what I meant to say. My remarks about colleges were not intended to cast any reflection upon the work they are doing. I know that not only the Harvard and Boston schools and many others are doing not only an immense amount of work of absolute charity, but in Boston they have thrown open the college infirmary so that men who are not students, that is, the profession at large, go there certain days, in the week and work for the poor people. What I meant to say was that I believe the dentist will have to be educated at the college; that he is under some civic and philanthropic obligations.

My friend sitting here beside me intimated that some of these people who attend the dental clinic should see the family dentist. He is quite right. There is always that danger that those who can afford to pay will take advantage of the free clinics. The medi al men are troubled about it too, but you can imagine that the families of the children we work among in New York, that a family dentist is a luxury that such families do not possess. If you can tide the children over a few years until they learn the value of the teeth, they will become the patients of some dentist in the future, and they will be able to pay for their work and will make more intelligent patients.

I thoroughly deplore the existence of dental parlors as a menace to the health of the community generally. There are various societies who have poor people applying to them, who are able to pay something, and who come to them and ask them for a dentist, or ask to be sent to a dentist who will not rob them, and they give the names of young men who have not a full practice, and let them take them for some small fee, and at such times as they are not busy, and it has worked very well, and quite a number of young men I know have been really tided over a critical period in their practice in this manner, and have been saved the possible misfortune of going to work for a dental parlor.

Dr. D. N. McQuillen:—I think it is only fair that it should be made known that our Director of Public Charities, Dr. Joseph Neff, is keenly alive to this question, and that he has it very much in his mind. He has told me so several times, and I have no doubt if we could get together, if this society would appoint a committee to take the matter up, they would get a great deal of help from him. There has been a great deal of work done in the public schools in the matter of eye examination, and I know the Director is very much interested in this question. I want to thank Drs. Potter and Wheeler for their most excellent papers.

Dr. Potter, closing:—We must try not to fall into a condition of despair. All great undertakings seem at certain stages to be nearly hopeless, and ours is no exception to the rule. We must, however, take courage, and do what we can, not expecting to do everything at once.

A word about dental dispensary fees. To my mind it is a great deal better to charge a small fee than to give services absolutely free. If a small fee is charged charitable work can be more widely extended, and will be better appreciated. There are, of course, cases of absolute need where even a small fee should be remitted.—*Dental Brief.*

